# Hot electron transfer in Zn–Ag–In–Te nanocrystal–methyl viologen complexes enhanced with higher-energy photon excitation†

**DOI:** 10.1039/d0ra02842h

**Published:** 2020-04-24

**Authors:** Tatsuya Kameyama, Kouta Sugiura, Susumu Kuwabata, Tomoki Okuhata, Naoto Tamai, Tsukasa Torimoto

**Affiliations:** Graduate School of Engineering, Nagoya University Chikusa-ku Nagoya 464-8603 Japan; JST, PRESTO 4-2-8 Hon-cho Kawaguchi Saitama 332-0012 Japan; Graduate School of Engineering, Osaka University Suita Osaka 565-0871 Japan; School of Science and Technology, Kwansei Gakuin University 2-1 Gakuen Sanda Hyogo 669-1337 Japan torimoto@chembio.nagoya-u.ac.jp

## Abstract

The dynamics of hot electron transfer from Zn–Ag–In–Te (ZAITe) nanocrystals (NCs) to adsorbed methyl viologen (MV^2+^) were investigated by transient absorption spectroscopy. The bleaching of the exciton peak in the ZAITe NC–MV^2+^ complexes evolved faster than that of ZAITe NCs. The hot electron transfer efficiency increased from 45% to 72% with increasing excitation photon energy.

## Abstract 2

Zn–Ag–In–Te nanocrystals exhibited hot electron transfer to adsorbed methyl viologen, the efficiency being enhanced from 45% to 72% with an increase in the excitation photon energy.

## Graphical abstract

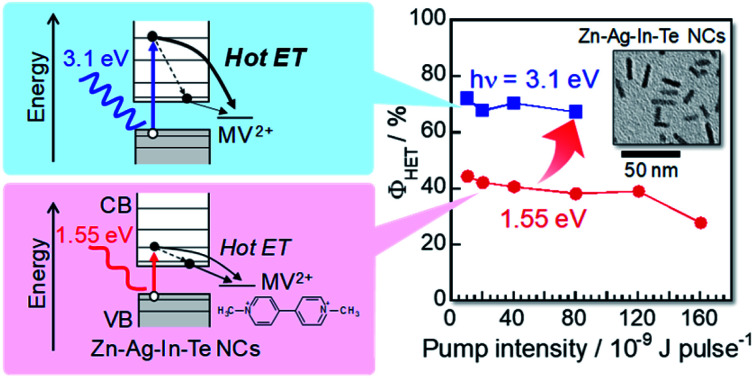

## Introduction

Tremendous efforts have been devoted to the development of highly efficient light-energy conversion systems, such as photovoltaics and photocatalysts, using size-quantized semiconductor nanocrystals (NCs).^1–4^ An understanding of the charge carrier extraction processes from NCs to species on their surface is prerequisite for improving the conversion efficiency. Charge separation dynamics with the use of various electron acceptors has been reported in many papers.^5–17^ For example Banin *et al.* reported that hybrid nanostructures of Au-tipped CdS nanorods with a larger Au size exhibited a higher probability for charge separation of multiple excitons formed in the CdS nanocrystals, improving the photocatalytic activity for H_2_ generation.^15^ The rate of photoinduced electron transfer from CdSe NCs to methyl viologen (MV^2+^) was shown to be dependent on the particle morphology: plate-like CdSe NCs exhibited a slower rate than that of spherical or rod-shaped CdSe NCs due to the weak electronic coupling along the short and long axes in the lateral dimension.^11^ Furthermore, various systems for efficiently utilizing hot carriers photogenerated in NCs have been proposed.^18,19^ Alivisatos *et al.* reported that PbSe NC arrays exhibited a transient photocurrent with a high excitation intensity, the current obtained being 6–9 orders of magnitude higher than a dark current due to the fast tunneling process of hot carriers produced *via* Auger recombination of multiple excitons.^20^ Zhu *et al.* reported shape-dependent hot electron transfer of CdSe NCs, in which the transfer rate from CdSe nanorods to MV^2+^ was faster than that obtained with spherical NCs.^21^

Recently, instead of using binary NCs showing high toxicity, such as CdSe, CdTe, and PbS, group I–III–VI semiconductor NCs have attracted increasing attention as light-absorbing materials with practical applications.^22–34^ For example, Zhong *et al.* reported that NCs composed of a Zn–Cu–In–Se solid solution, showing an absorption onset around 860–1060 nm, acted as efficient sensitizers for porous TiO_2_ electrodes and that the resulting sensitized solar cells exhibited solar energy conversion efficiency of 11.6%.^28^ We reported the preparation of Zn–Ag–In–Te (ZAITe) NCs with a rod shape, the energy gap (*E*_g_) of which was enlarged from 1.20 to 1.60 eV with an increase in the Zn content.^27,32,34^ The obtained ZAITe NCs exhibited a narrow band-edge photoluminescence (PL) peak with a high quantum yield of *ca.* 47%. The PL peak wavelength was tunable in the near-IR wavelength region depending on their chemical composition.^32^ Furthermore, continuous light irradiation to ZAITe NC monolayers immobilized on ITO electrodes produced a cathodic photocurrent at an electrode potential more positive than that of their valence band edge, and then the observed photocurrent intensity increased with irradiation of higher-energy photons, indicating hot hole injection from ZAITe NCs to ITO electrodes.^34^

By using transient absorption spectroscopy, the migration of hot carriers in NCs can be directly observed, enabling better clarification of the photochemical properties of I–III–VI-based NCs. However, compared to studies on conventional binary NCs, there have been few studies on charge extraction dynamics, especially for hot electrons photogenerated in I–III–VI-based NCs. In this study, using methyl viologen (MV^2+^) as an electron acceptor, we prepared ZAITe NC–MV^2+^ complexes to evaluate the dynamics of hot electron transfer from ZAITe NCs to MV^2+^ under excitation of light with various photon energies.

## Experimental

**Fig. 1:**
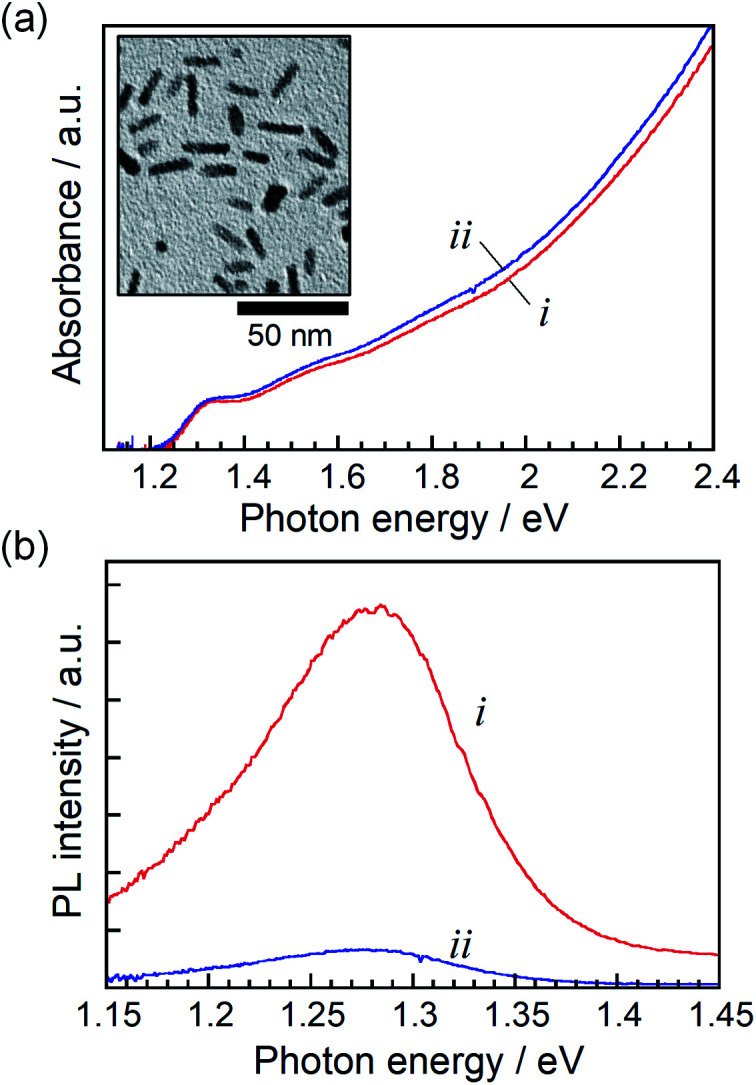
Steady-state absorption (a) and PL (b) spectra of ZAITe NCs (i) and ZAITe NC–MV^2+^ complexes (ii). Excitation photon energy was 3.4 eV. The inset in (a) shows a typical TEM image of ZAITe NCs.

Rod-shaped nanocrystals of a ZAITe solid solution were synthesized by the reaction of metal acetate and trioctylphosphine telluride in 1-dodecanethiol, as reported in previously.^32^ Thus-obtained NCs had a dimension of 3.9 ± 0.5 nm in width and 18.3 ± 1.5 nm in length (inset of Fig. 1a), the chemical composition of which was *x* = 0.75 in the chemical formula of (AgIn)_*x*_Zn_2(1−*x*)_Te_2_. ZAITe NC–MV^2+^ complexes were prepared by adding a drop of MV^2+^ ethanol solution to an octane solution containing ZAITe NCs (The detailed procedure is described in ESI.†) according to a previous report on CdSe–MV^2+^ complexes.^11,21^ The average number of MV^2+^ molecules adsorbed on a ZAITe NC was estimated to 16. A further increase in the number of adsorbed MV^2+^ molecules in a complex resulted in the aggregation of ZAITe NC–MV^2+^ complexes due to the decrease in solubility of ZAITe NCs in octane.

## Results and discussion

Fig. 1 shows steady-state absorption and PL spectra of ZAITe NCs and ZAITe NC–MV^2+^ complexes uniformly dispersed in octane. The absorption spectrum of ZAITe NC–MV^2+^ exhibited the absorption onset around 1.27 eV and the exciton peak at 1.33 eV, being in good agreement with those observed for ZAITe NCs only. The *E*_g_ was estimated from the absorption onset to be 1.27 eV. It should be noted that the absorption peak of MV^2+^ appeared in the higher energy region (>3.88 eV) and could not be obstructive of measuring the spectra of ZAITe NCs. ZAITe NCs show a band-edge emission with a relatively narrow peak width in the near-infrared region. As shown in Fig. 1b, the PL intensity was remarkably quenched by the adsorption of MV^2+^ molecules on ZAITe NCs, indicating photoinduced electron transfer from ZAITe NCs to MV^2+^. We previously reported that the level of the conduction band minimum (*E*_CB_) of ZAITe NCs was −1.3 V *vs.* Ag/AgCl for the composition of *x* = 0.75.^34^ Since the redox potential of MV^2+^/MV˙^+^, −0.65 V *vs.* Ag/AgCl,^35^ was more positive than the *E*_CB_ of ZAITe NCs used, excited electrons in the present ZAITe NCs were able to efficiently transfer to MV^2+^.

We measured the transient absorption (TA) spectra of ZAITe NCs and ZAITe NC–MV^2+^ complexes excited by 1.55 eV photons, the excitation photon energy (*hν*) being a little higher than the *E*_g_ of ZAITe NCs, *hν*/*E*_g_ = 1.22. Since TA dynamics obtained with the excitation intensities of 20 × 10^−9^ and 40 × 10^−9^ J per pulse gave almost the same decay profiles (Fig. S1†), Auger recombination of multiple excitons could not be observed with the light intensities used in the present study, proving that the excitation intensity used, 40 × 10^−9^ J per pulse, was low enough to only produce single excitons in NCs.

**Fig. 2:**
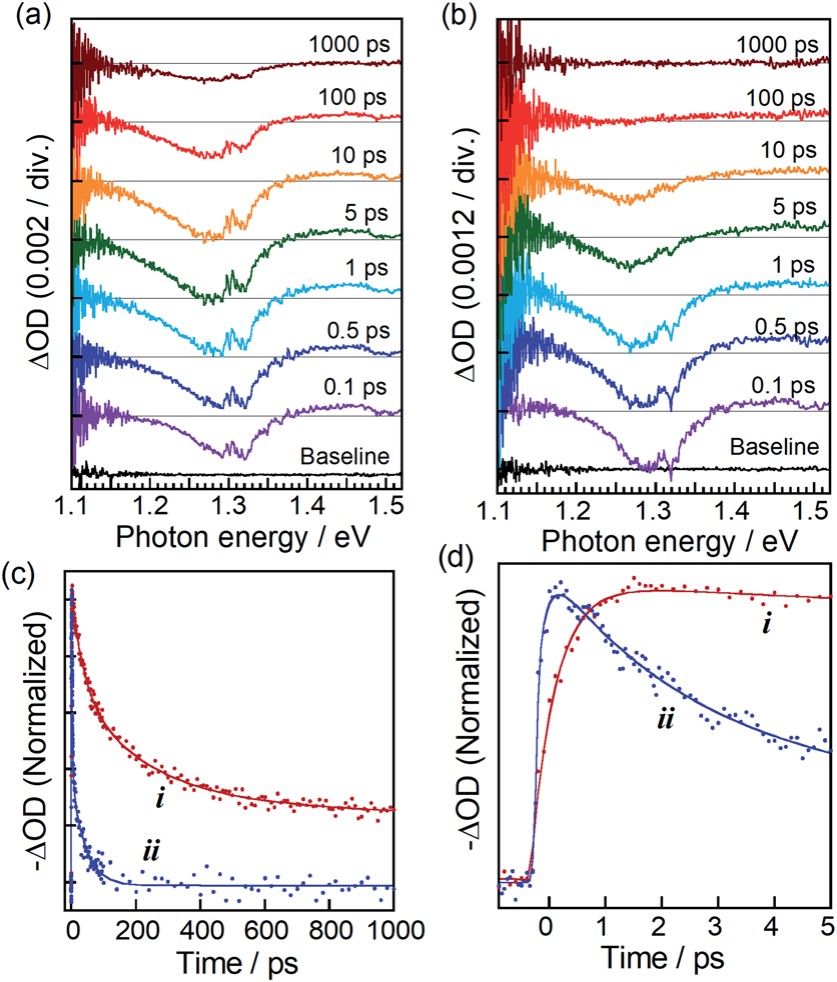
(a and b) Transient absorption spectra of ZAITe NCs (a) and ZAITe–MV^2+^ complexes (b) excited by irradiation of 1.55 eV photons with pump intensity of 40 × 10^−9^ J per pulse. (c) TA recovery dynamics at 1.29 eV of ZAITe NCs (i) and ZAITe NC–MV^2+^ complexes (ii). (d) An enlarged view of panel (c) with time of less than 5 ps.

TA spectra in Fig. 2a and b exhibited a bleaching peak at about 1.29 eV assignable to the state filling of the lowest energy exciton in ZAITe NCs, regardless of the presence of MV^2+^. However, the peak recovery rate was significantly shortened by the MV^2+^ adsorption. It should be noted that a TA peak of MV˙^+^ radial at about 1.85 eV, representing the electron transfer process from ZAITe NCs to MV^2+^, could not be accurately detected in this study because of the weak transient absorption signal of MV˙^+^ radical at the low excitation intensity of 40 × 10^−9^ J per pulse used in the experiment.

Fig. 2c and d show the time profiles of bleaching peak signals at 1.29 eV of ZAITe NCs and ZAITe NC–MV^2+^ complexes. Although the TA spectral feature of the ZAITe NC–MV^2+^ complexes was very similar to that of ZAITe NCs only, the bleaching peak of ZAITe NC–MV^2+^ complexes rapidly disappeared within 100 ps, the recovery time being much faster than that observed for ZAITe NCs, >1 ns. This was due to the capture of electrons in the conduction band edge state of ZAITe NCs with adsorbed MV^2+^. Interestingly, it can be clearly seen in Fig. 2d that the bleaching peak was much more rapidly evolved for ZAITe NC–MV^2+^ complexes than for ZAITe NCs. We analysed the ultrafast dynamics of TA signal evolution within *ca.* 1 ps by fitting a multiexponential function (Table S1†). The obtained rise-time was shortened by the complex formation: the rise-times of ZAITe NCs and ZAITe NC–MV^2+^ complexes, *τ*_rise_(ZAITe) and *τ*_rise_(ZAITe–MV^2+^), were 450 fs and 220 fs, respectively. Similar rise-time shortening was reported for CdSe NC–MV^2+^ complexes^9,36^ and CdSe–Au hetero NPs^17^ as direct evidence for the transfer of hot electrons from NCs to acceptors, MV^2+^ or Au NPs, respectively. Furthermore, it was reported that hot electrons in CdSe NCs were relaxed to the band-edge state with a lifetime of a few hundred femtoseconds to several picoseconds, being comparable to the time range of TA signal evolution shown in Fig. 2d. Thus, since we could assume that *τ*_rise_(ZAITe–MV^2+^) was expressed as the inverse of the sum of rate constants of intraband relaxation and hot electron transfer, the rise-time shortening by the complex formation clearly indicated the extraction of hot electrons in ZAITe NCs with adsorbed MV^2+^. Thus, we estimated the hot electron transfer efficiency (*ϕ*_HET_) with 1.55 eV photon excitation to be *ca.* 51% by the following equation:^17^*ϕ*_HET_ (%) = {1 − *τ*_rise_(ZAITe–MV^2+^)/*τ*_rise_(ZAITe)} × 100.

**Fig. 3:**
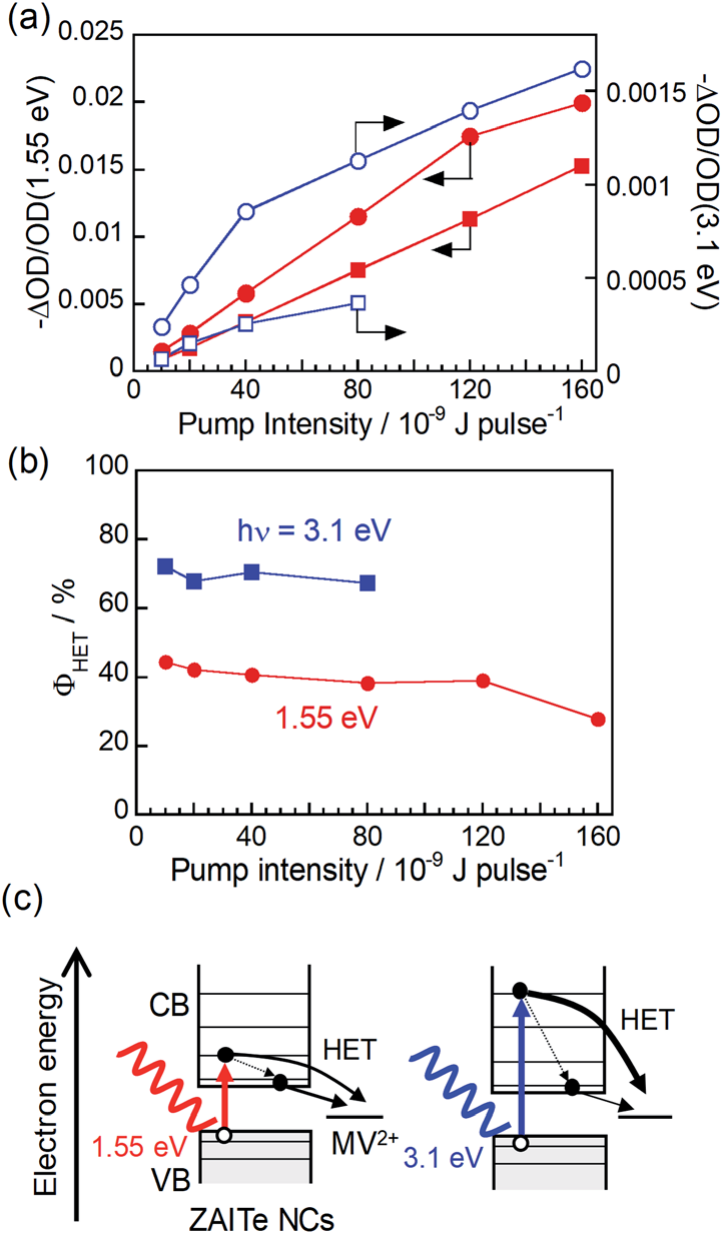
(a) Initial amplitudes of transient bleach signals at 1.29 eV produced by excitation with 1.55 eV (solid symbols) and 3.1 eV photons (open symbols) as a function of pump intensity. The samples were ZAITe NCs (circles) and ZAITe NC–MV^2+^ complexes (squares). (b) *ϕ*_HET_ with excitation of 1.55 eV and 3.1 eV photons as a function of pump intensity. (c) Schematic illustration of hot electron transfer after excitation with different photon energies.

We calculated the ratio, −ΔOD/OD(1.55 eV), of the initial amplitude of transient bleaching signal at the band-edge state (1.29 eV) (Fig. S2†) to the absorbance of the grand state at the excitation photon energy of 1.55 eV in order to normalize the difference in the number of absorbed photons used for excitation. The results for ZAITe NCs and ZAITe–MV^2+^ complexes are plotted in Fig. 3a as a function of pump intensity. The −ΔOD/OD(1.55 eV) ratio was remarkably decreased by complex formation with MV^2+^ regardless of the pump intensity. This indicated that the number of photogenerated electrons that relaxed to the conduction band-edge state became smaller for ZAITe–MV^2+^ complexes, owing to the extraction of hot electrons with MV^2+^. The value of *ϕ*_HET_ can also be estimated by the following equation:^17^*ϕ*_HET_ (%) = {1 − (ΔOD_ZAITe–MV^2+^_/ΔOD_ZAITe_)} × 100.

The results are shown in Fig. 3b. With a decrease in pump intensity to 1.0 × 10^−9^ J per pulse, the *ϕ*_HET_ value slightly increased to 45%, even though the excitation photon energy of 1.55 eV was modestly larger than *E*_g_ of ZAITe NCs used, 1.27 eV. It has been reported for CdSe–Au hybrid NPs in our previous paper^17^ that the ultrafast electron transfer occurred from the conduction band edge state of CdSe NPs to the attached Au NPs, resulting in that the *ϕ*_HET_ values obtained from the TA rise-time analysis were significantly smaller than those simply estimated from the bleaching signal analyses, that is, {1 − (ΔOD_CdSe–Au_/ΔOD_CdSe_)} × 100. However, in the present study, the obtained *ϕ*_HET_ values were very similar between the analyses of TA rise-times and bleaching signals, indicating that such an ultrafast electron transfer from the band-edge state hardly contained in the charge transfer processes in ZAITe NC–MV^2+^ complexes.

Furthermore, we evaluated the dependence of *ϕ*_HET_ on the excitation photon energy. By analysing TA spectra obtained with excitation of 3.1 eV photons, the ratios of −ΔOD/OD(3.1 eV) are also plotted as a function of pump intensity as shown in Fig. 3a. The values of −ΔOD/OD were more significantly decreased with the complexation with MV^2+^ in the case of excitation of 3.1 eV photons in comparison to those of 1.55 eV photons. The excitation with 3.1 eV photons gave the *ϕ*_HET_ of 72% with pump intensity of 1.0 × 10^−9^ J per pulse, being much larger than that obtained by 1.55 eV photon excitation, 45%. It was reported in our previous paper that multiple excitons could not be generated by the absorption of a single 3.1 eV-photon with a ZAITe NC despite an *hν*/*E*_g_ ratio of 2.44.^34^ Thus, the present results indicated that hot electrons, excited to a higher level with higher-energy photon absorption, could be more efficiently extracted with MV^2+^ (Fig. 3c). Similar behaviour has been observed for hot hole transfer from ZAITe NCs, in which the efficiency of cathodic photocurrent generation of a ZAITe NC monolayer was more significantly enlarged with an increase in the excitation photon energy from *ca.* 2.5 eV, owing to the increase in the probability of hot hole transfer from ZAITe NCs to the contact electrode. Furthermore, it should be noted that the *ϕ*_HET_ of 72%, observed for ZAITe NC–MV^2+^ complexes, was comparable to those reported for CdSe naonrod–MV^2+^ complexes, >50%,^7^ and for CdSe–Au hybrid NPs, 48%.^17^ This suggests that ZAITe NCs are a promising material to utilize the excess energy of incident photons for improving the conversion efficiency of quantum dot solar cells.

## Conclusions

We successfully clarified the electron extraction dynamics of ZAITe NCs with adsorbed MV^2+^ by femto-second transient absorption spectroscopy. Fast evolution of the bleaching peak, assignable to the state filling of the lowest energy exciton in ZAITe NCs, was observed due to the extraction of hot electrons in ZAITe NCs to adsorbed MV^2+^. Furthermore, hot electron transfer efficiency increased from 45% to 72% with an increase in the excitation photon energy from 1.55 to 3.1 eV. With a better understanding of the hot carrier extraction process of I–III–VI semiconductor NCs, we will fabricate light energy conversion systems in which the excess energy of high-energy photons absorbed can be utilized more efficiently.

## Conflicts of interest

There are no conflicts to declare.

## Supplementary Material

RA-010-D0RA02842H-s001
