# Supplementary material for: Hot electron transfer in Zn–Ag–In–Te nanocrystal–methyl viologen complexes enhanced with higher-energy photon excitation
Source: RSC Adv. 2020 Apr 24;10(28):16361–5. doi: 10.1039/d0ra02842h (PMC9052877; doi:10.1039/d0ra02842h)
Supplement: RA-010-D0RA02842H-s001 [file RA-010-D0RA02842H-s001.pdf]

Electronic Supplementary Material (ESI) for RSC Advances.
This journal is © The Royal Society of Chemistry 2020

Supporting information

Hot Electron Transfer in Zn-Ag-In-Te Nanocrystal-Methyl Viologen Complexes Enhanced with Higher-Energy Photon Excitation

Tatsuya Kameyama,[ab] Kouta Sugiura,[a] Susumu Kuwabata,[c] Tomoki Okuhata,[d] Naoto Tamai,[d] and Tsukasa Torimoto*[a]

[a]Graduate School of Engineering, Nagoya University, Chikusa-ku, Nagoya 464-8603, Japan.
[b]JST, PRESTO, 4-2-8 Hon-cho, Kawaguchi, Saitama 332-0012, Japan.
[c]Graduate School of Engineering, Osaka University, Suita, Osaka 565-0871, Japan.
[d]School of Science and Technology, Kwansei Gakuin University, 2-1 Gakuen, Sanda, Hyogo 669-1337, Japan.

Correspondence to: torimoto@chembio.nagoya-u.ac.jp

1. Experimental

Preparation of Zn-Ag-In-Te (ZAITe) NC-MV^{2+} complexes

ZAITe NC−MV^{2+} complexes were prepared according to a previous report on CdSe NC-MV^{2+} complexes with slight modification.[1,2] ZAITe NCs ($(AgIn)_xZn_{2(1-x)}Te_2$) in octane solution were synthesized with an x value of 0.75 by the reported method.[3] The original solution of ZAITe NCs (0.20 cm^3) was diluted ten times with octane, and then 10 mm^3 MV^{2+} (1.0 mmol dm^{-3}) of ethanol solution was added to the solution with vigorous stirring. After ethanol had been removed by evaporation, the solution was diluted to 5.0 cm^3 with octane. The sample of thus-prepared ZAITe NC-MV^{2+} complexes was used for measurement.

2. Results

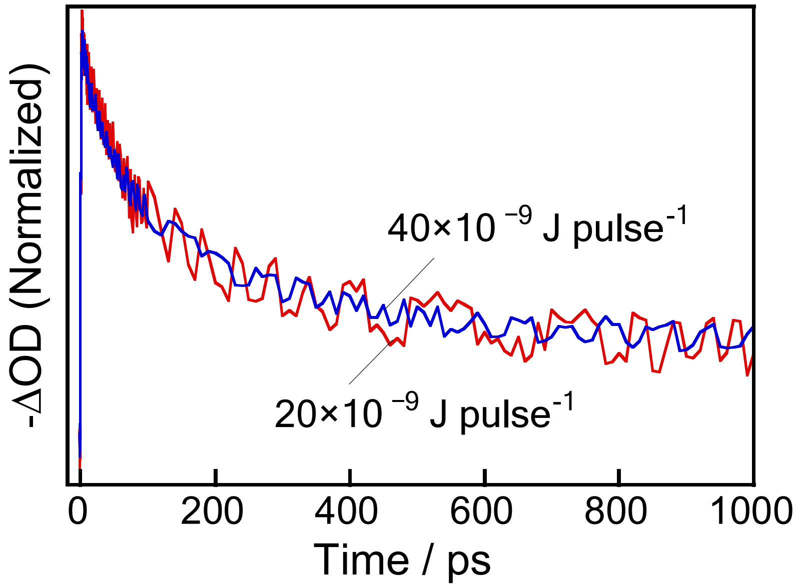

Figure S1. Comparison of TA dynamics of ZAITe NCs at 1.29 eV photoexcited by 1.55-eV photons with different excitation intensities.

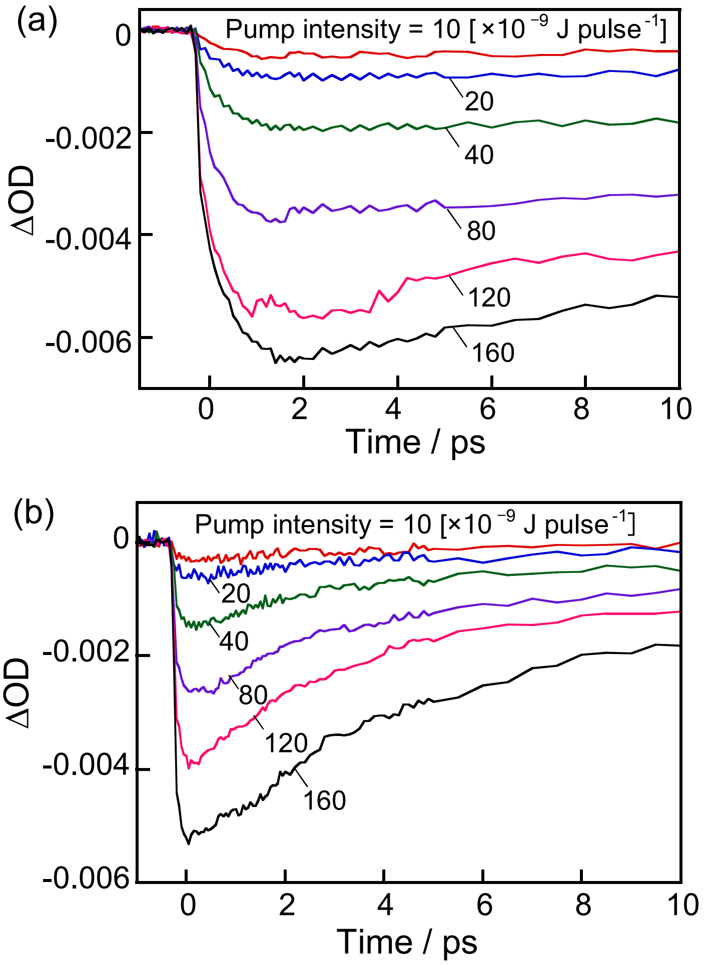

Figure S2. TA recovery dynamics of ZAITe NCs at 1.29 eV excited by irradiation of 1.55-eV photons with different pump intensities. The samples were ZAITe NCs (a) and ZAITe NC-MV^{2+} complexes (b). Profiles for the fast component time region of less than 10 ps are shown.

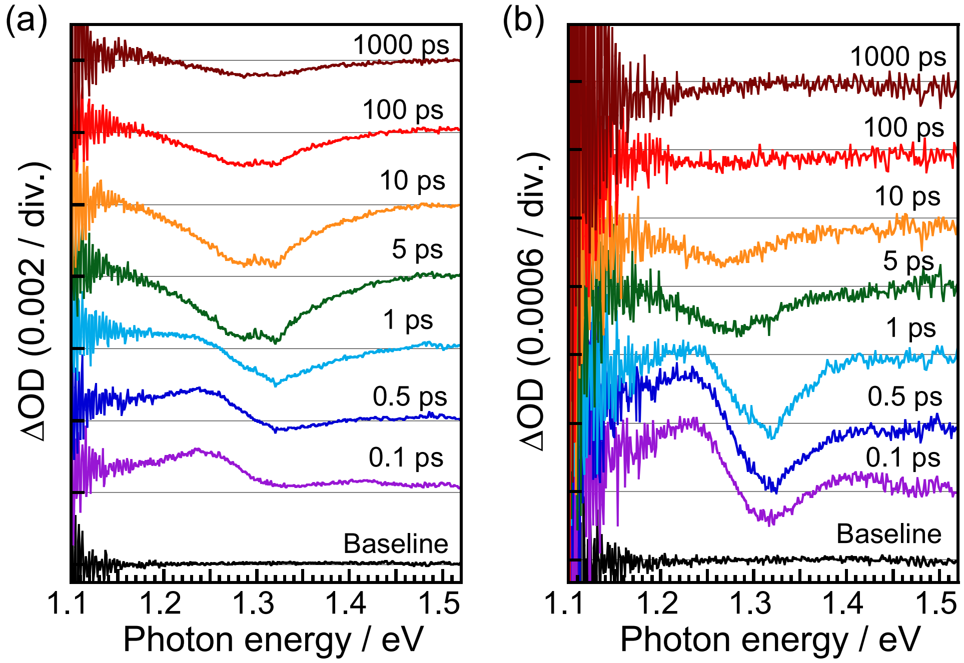

Figure S3. Transient absorption spectra of ZAITe NCs (a) and ZAITe NC-MV^{2+} complexes (b) excited by irradiation of 3.1-eV photons with pump intensity of 20×10^{-9} J $pulse^{-1}$.

Table S1. Multi-exponential fitting results for evolution and decay profiles of the bleaching signal at 1.29 eV in TA spectra (Fig. 2c and d).

Excitation energy / eV	Sample	hν/E_g	τ_{rise} / ps	τ_1 / ps (A_1/%)	τ_2 / ps (A_2/%)	τ_3 / ns (A_3/%)
1.55	ZAITe NCs	1.22	0.45	36 (31%)	210 (41%)	6.1 (28%)
	ZAITe NC-MV^{2+} complexes		0.22	2.7 (67%)	43 (33%)	

The TA decay profile can be fitted with the form, $\Delta OD(t) \propto \sum_i A_i exp(-\frac{t}{\tau_i})$, where τ_i represent the lifetimes and A_i are the corresponding amplitudes.[4] The measured decay parameters along with the rise-time (τ_{rise}) are also presented. For obtaining the best-fit curve, tri- and bi-exponential functions were applied for ZAITe NCs and ZAITe NC-MV^{2+} complexes, respectively.

3. References

[1] Zhu, H.; Lian, T.; *J. Am. Chem. Soc.* **2012**, *134*, 11289−11297

[2] Okuhata, T.; Tamai, N.; *J. Phys. Chem C*, **2016,** *120,* 17052−17059

[3] Kameyama, T.; Sugiura, K.; Ishigami, Y.; Yamamoto, Y.; Kuwabata, S.; Okuhata, T.; Tamai, N.; Torimoto, T.; *J. Mater. Chem. C*, **2018**, *6*, 2034−2042

[4] Wang, Y. F.; Wang, H. Y.; Li, Z. S.; Zhao, J.; Wang, L.; Chen, Q. D.; Wang, W. Q.; Sun, H. B.; *J. Phys. Chem. C*, **2014**, *118*, 17240−17246
